# Advanced Methodology and Preliminary Measurements of Molecular and Mechanical Properties of Heart Valves under Dynamic Strain

**DOI:** 10.3390/ijms21030763

**Published:** 2020-01-24

**Authors:** Rama S. Madhurapantula, Gabriel Krell, Berenice Morfin, Rajarshi Roy, Kevin Lister, Joseph P.R.O. Orgel

**Affiliations:** 1 Department of Biology, Illinois Institute of Technology, Chicago, IL 60616, USA; bmorfin@hawk.iit.edu; 2 Pritzker Institute of Biomedical Science and Engineering, Illinois Institute of Technology, Chicago, IL 60616, USA; gkrell1@hawk.iit.edu; 3 Corvid Technologies, Mooresville, NC 28117, USA; rajarshi.roy@corvidtec.com (R.R.); kevin.lister@corvidtec.com (K.L.); 4 Department of Biomedical Engineering, Illinois Institute of Technology, Chicago, IL 60616, USA

**Keywords:** heart valves, tissue transition regions, stress–strain relations, X-ray diffraction scanning, valve failure

## Abstract

Mammalian heart valves are soft tissue assemblies with multi-scale material properties. This is because they are constructs comprising both muscle and non-contractile extracellular matrix proteins (such as collagens and proteoglycans) and transition regions where one form of tissue structure becomes another, significantly different form. The leaflets of the mitral and tricuspid valves are connected to chordae tendinae which, in turn, bind through papillary muscles to the cardiac wall of the ventricle. The transition regions between these tissue subsets are complex and diffuse. Their material composition and mechanical properties have not been previously described with both micro and nanoscopic data recorded simultaneously, as reported here. Annotating the mechanical characteristics of these tissue transitions will be of great value in developing novel implants, improving the state of the surgical simulators and advancing robot-assisted surgery. We present here developments in multi-scale methodology that produce data that can relate mechanical properties to molecular structure using scanning X-ray diffraction. We correlate these data to corresponding tissue level (macro and microscopic) stress and strain, with particular emphasis on the transition regions and present analyses to indicate points of possible failure in these tissues.

## 1. Introduction

It is estimated that ∼11.7 million people in the United State of America suffer from heart disease [1]. A major burden on physicians is in analyzing risk factors and identifying potential markers of future valve failure during routine health checkups [2]. A major improvement in early diagnosis could reduce the burden on the healthcare system and the patients alike, and also improves post-surgery prognosis. To thoroughly understand valve failure, it is crucial to understand the material properties of heart valves and their responses to extreme mechanical strain as observed in heart valve failure. This information, in conjunction with observations on vital signs (for instance blood pressure, heart murmurs) and medical and family history may prove useful to early diagnosis of potential valve failure. However, these material properties and responses are not as well defined, currently. The purpose of this current work is to present an enhanced research methodology to make it possible to determine the relationship between molecular structural changes and larger scale biomechanical properties.

### 1.1. Heart Valve Tissue Organization

**Figure 1:**
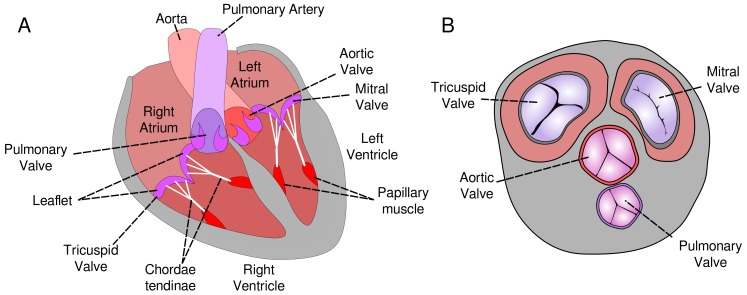
Illustration of the human heart valves. The leaflet of the MV and TV is held in position by CT that extend into the PM, which attach on to the wall of the heart. (**A**) Lateral (frontal) cross-section, (**B**) Transverse cross-section.

Human heart valves act as gatekeepers to help ensure unidirectional flow of blood under physiological conditions. Because of their different placements, they control blood flow in the pulmonary and systemic circuits and between the atria and ventricles. The four main valves experience significantly different blood pressures and rates of activation. Blood flow/pressure activates the valves to allow the flow of blood away from a chamber of the heart and they close to prevent blood flow in the wrong direction. The aortic valve (AV) allows the outflow of blood from the left ventricle to the aorta and the pulmonary valve (PV) controls the outflow of blood from the right ventricle to the pulmonary artery (Figure 1). These valves are somewhat simple in architecture consisting of three leaflets or cusps that attach to the wall of the valve. In contrast, the mitral (bicuspid; MV) and the tricuspid (TV) valves control the outflow of blood from the right and left atria into ventricles respectively and are more complicated in both structure and function. The leaflets (LL) of these valves are connected to chordae tendinae (CT) which are responsible for providing the support from the leaflets to fold over themselves through the normal heart cycle. The CT are in turn connected to papillary muscles (PM) and finally to the wall of the ventricle.

### 1.2. Heart Tissue Organization and Cardiac Injury

As is clear from visual inspection, the MV and TV have two distinct tissue transitions, where one specialized tissue organizational domain becomes another with a different organization, even though they are principally made of the same materials; namely (i) LL to CT (one type of fibrillar collagen and proteoglycan (PG) arrangement to another; see SI Section S1.2 for more details) and (ii) CT to PM (chiefly fibrillar collagen and PG to skeletal-like cardiac muscle) [3,4,5]. As one would expect, these transition regions exhibit markedly different material and mechanical properties than at either end of the transition where the tissue is ‘pure’ LL, CT or PM. In structure, PM resembles skeletal muscles and the cellular arrangement is markedly different from the packing exhibited by cardiac muscles, as indicated by the average sarcomere length [6,7,8]. For more information regarding comparisons of sarcomere lengths, see SI Table S1. The LL itself consists of various regions and layers of different types of materials [9]. These layers in the LL lead to collagen molecular packing/fiber arrangement structures that are markedly different from the alignment and packing in the CT. The additional layers and alignment of fibers therein allow for expansion of the LL for valve closure and opening (see SI Figure S3) [9].

One may expect, a priori, some manner of muscle-to-tendon transition in cardiac valves, analogous to that found in skeletal tissues. So, the mechanism of injury to the latter may be beneficial for determining similar responses in the former. Previous studies have demonstrated that injury to tissues that possess muscle-to-tendon transitions originates in those transition regions [10,11,12]. Recently, it has been shown that the transition between muscle to tendon is complex and diffuse, exhibiting large differences in biomechanical properties within a small range of the fibrous tissue’s overall length [13]. Strain-based injury to skeletal muscles frequently occurs in the muscle–tendon transition region, within which muscle fibers detach from collagen fibrils [12]. Similarly, it has been reported that traumatic injury to the heart often causes tears in the CT of cardiac valves [14] and are also observed to rupture after a prolapse of the mitral valve [15]. In some cases, mitral valve prolapse has been associated with an abnormal collagen content in the LL and CT [16]. Considering the significant advances in cardiac therapy and surgery in recent decades, it is reasonable to suppose that any progress in understanding hitherto under-characterized mechanical properties of valvular material dysfunction may be helpful to further advancements in valve repair and replacement.

### 1.3. Data-Based Models for Diagnosis, Treatment and Prevention of Valve Injury

Importantly, these data are needed to develop high definition computational models of the heart that are intended to closely reflect physiological function. With the development of high-speed computing and modeling techniques, cardiac flow simulations have made it possible to establish potential cardiac injury mechanisms in simulations. These simulations can aid in predicting cardiac damage, particularly chordal tear and valvular defects [17,18,19,20]. Correlations between fluid dynamics studies on heart valves and physiological measurements, such as blood pressure (BP), electrocardiograph (ECG), trans-thoracic echo cardiograph (TTE) are becoming instrumental in diagnosing cardiac defects, and injuries. They are also being used to develop new surgical techniques to improve prognosis for patients undergoing valve repair or replacements [21,22,23].

A major hurdle in improving the quality and reliability of simulations focused on the function and injury in heart valves is the lack of relatively high-resolution data (sub-micron to nanometer scale) concerning tissue composition, tissue boundaries, and elastic properties of heart valves. Several modeling efforts have been focused on developing the elastic contributions of the various elements of a tissue assembly [24,25,26,27,28,29,30,31]. Few efforts have laid particular importance on the transition regions between constituent tissues. For instance, Roux et al. (2016) [32] and Sharafi et al. (2011) [33] have previously developed models of the muscle–tendon junction. It is of utmost importance to establish these interfacial material and mechanical properties in order to be able to develop a higher definition model of heart valves. This task is particularly more complicated because of the aforementioned nature and properties of these regions. Madhurapantula et al. (2017) [13] showed that the material properties, such as the Young’s modulus, varied considerably at different points over the range of a few mm of skeletal muscle without visible differences in the material composition of the tissue transition region as viewed by light microscopy. However, by using XRD Madhurapantula et al. (2017) [13] were able to track changes in material composition (muscle vs. collagen relative content) along the transition region without introducing artifacts to the sample, on a sub-mm scale. In addition, the study recorded molecular elongation (hereinafter, “molecular strain”) through characterizing XRD of muscle and collagen respectively. The ability to track macroscopic strain (hereinafter, “engineering strain”) in relation to stress and molecular strain together appears to be a potentially powerful tool to the study of cardiac valve stress–strain response and provide insights into the regions of the valve tissue that appear most vulnerable to injury on the basis of their mechanical and molecular response to strain. See Section 4.4 for definitions of molecular strain for tissues.

We present here expanded methods and analyses to establish material and mechanical properties of cardiac tissues, mitral and tricuspid valve transition regions. These methods are an advancement of those described in Madhurapantula et al. (2017) [13] that were applied to relatively simple, single transition region changes from tendon to skeletal muscle. Here, we take advantage of developments in improved data collection and analysis to address more complex sub-tissues within cardiac valves, while comparing and contrasting their apparent structure and mechanical properties at both the molecular and microscopic scale simultaneously. For each sample under the application of stretch, specific areas of the samples were tracked through microscopic measurements in addition to correlating these changes with the molecular composition and strain changes using scanning XRD. Multiple diffraction exposures were tracked and correlated to scan the sample using the X-ray beam as the probe, similar in technique to scanning microscopy [34]. These XRD data were used to gain molecular level insights to each part of the cardiac valve tissue after application of stretch. We report (i) XRD scanning data to determine internal tissue boundaries and composition for the LL–CT and CT–PM transitions in pig MV and TV; (ii) XRD data to determine collagen d-spacing changes along these transitions to calculate molecular strain that appear to indicate points of potential molecular and therefore bulk material failure, and; (iii) microscopic evaluation of the local regional strain and isolated tissue component strain(s) in these tissues. This information can now be used to improve computational models and provide important insights into early-stage diagnostic criteria for valve failure.

## 2. Results

### 2.1. Tissue Composition Along Transition Regions

**Figure 2:**
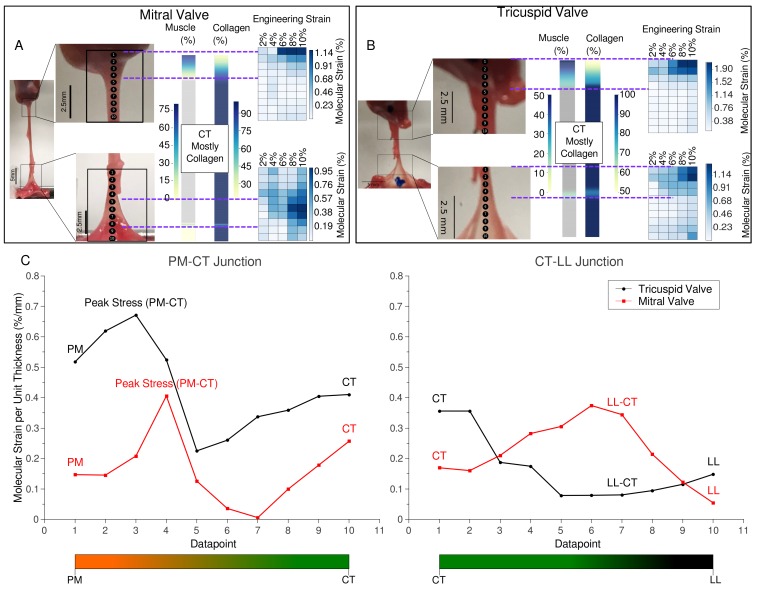
Tissue composition and molecular strain vs. applied engineering strain maps from ‘bisected’ MV and TV samples. (**A**,**B**) Composition maps showing muscle and collagen percentages along the PM–CT and CT–LL junctions. These transitions are diffuse in nature. Particularly in the PM–CT junction, the CT extends into the PM by a few millimeters. There is a near-linear decrease in collagen percentage, going further into the PM, starting at the beginning of the PM. These transition regions are marked by the purple dotted lines. A comparison of engineering strain (applied stretch/strain) vs. the molecular strain (calculated from changes in collagen D-period elongation) is shown as heatmaps for both the transitions from MV and TV. An increase in molecular strain is observed, particularly in the PM–CT junction. Similar localization of molecular strain is observed in the LL–CT transition of the MV but not the TV. (**C**) Tracking of molecular strain per unit thickness along the PM–CT and the CT–LL transition regions of MV and TV. The molecular strain data are obtained from the 10% engineering strain series in each case. This measure provides with a representation of local strain distribution, which may be helpful to determine the point of breakage. As observed, the PM–CT junction assumes highest molecular strain (peak stress labeled on the plot). A second potential point of failure is observed in the MV, at the LL–CT transition. However, the overall molecular strain per unit length experienced by the LL–CT junction is lower than that of the PM–CT.

XRD scanning was performed along the samples dissected from pig valves. Diffraction patterns were analyzed to identify and measure the collagen fifth-order meridional and muscle (1,1) equatorial peaks. The relative intensity of these peaks was used to calculate the relative percentage composition of muscle and collagen along the transition regions (Figure 2), as per Madhurapantula et al. (2017) [13]. Note that these are not absolute percentage composition values, but percentage muscle relative to collagen content to give a relative comparison of the two major load-bearing components of the tissue.

MV and TV have clear differences in mechanical properties, evident also from consideration that one is a bi-part and the other a tri-part valve. However, the material composition from the respective muscle to collagenous tissue transitions are similar. As noted in the study of skeletal muscle to tendon molecular composition transition is not a ‘step-change’, but is diffuse or gradual over a section of the fibrous tissue. XRD data shows a near-linear decrease in collagen percentage is observed in the PM to CT transition, going into the belly of CT (no muscle detected), the length of this transition spanning a few millimeters.

The transition between CT–LL, as determined by XRD, appears to be somewhat more homogeneous than the PM–CT transition. This is due to fact that the LL and CT are both primarily constituted of fibrillar collagen [9], unlike the PM–CT junction as demonstrated here, and in Madhurapantula et al. (2017) [13] (XRD detects muscle and/or collagen diffraction and therefore material composition of that sample region, and thus are estimated to be one or the other as the principal tensile loading component of that tissue section). There appears to be a second series of collagen fibers, aligned at 90° to the axis of the CT, at the apparent, visible junction between CT and LL. This marks a discernibly different part of the tissue transition region that is distinct from pure CT and the rest of the CT–LL transition area.

The AV is distinctly different from the MV and TV, consisting of three cusps attaching to the wall of the aorta (see Figure 1). Composition and molecular strain maps for AV are reported in SI Section S3.2.

### 2.2. Changes in Molecular vs. Engineering Strain with Application of Stretch to ‘Bisected’ Samples (Observations within Transition Regions)

MV and TV samples were cut and separated, halfway along the CT (‘bisected’) to capture stress vs. strain properties of these segments. XRD scans along these ‘bisected’ samples were used to determine changes in the molecular packing structures of muscle and collagen along the tissues (Figure 2).

Molecular strain was calculated as the change in the length of the collagen D-period from that point recorded at ‘Resting’ (minimum load; approximately ~0.1 g) state as described in Madhurapantula et al. (2017) [13]. Ten data points were recorded along the length of the sample and were tracked as their position changed with increasing strain. These data are reported against corresponding engineering strain (applied stretch) as a heatmap (Figure 2).

The PM region is most compliant and the greatest strain can be observed in this element in the PM–CT transition in both MV and TV. The molecular strain is highest at around the PM–CT transition, where the corresponding composition scan shows a steep increase in collagen content in the tissue (see Section 2.1). This strain drops significantly at the end of the PM in the PM–CT transition, i.e., in the CT region, demonstrating a localization of strain at the bottom of the PM–CT junction and a gradient of strain going into the PM.

A similar trend of localization of molecular strain is observed in the CT–LL transition of the MV and TV. Strain is localized at the point in the transition where there appears to be a muscle-like diffraction series. This can be observed using the corresponding composition scans.

Figure 2C shows a relationship between molecular lengthening of collagen fibers per unit thickness of the valve section sample at 10% engineering strain. It seems noteworthy, that the highest point of molecular strain per unit thickness is in the PM–CT transition of both valves (around point 3–4), possibly indicating a point of vulnerability in relation to mechanical failure. Although at a lower strain value, which may be understandable considering the greater relative concentration of comparatively stiff collagen relative to muscle, the CT–LL transition regions also indicate possible failure vulnerability; the mitral valve at around points 1–2 and tricuspid at around 5–7, although these possible indicators are significantly less pronounced than that of the PM–CT transition and thus may be less significant.

### 2.3. Changes in Local Collagen Fiber Orientation in the LL–CT Junction with the Application of Stretch

**Figure 3:**
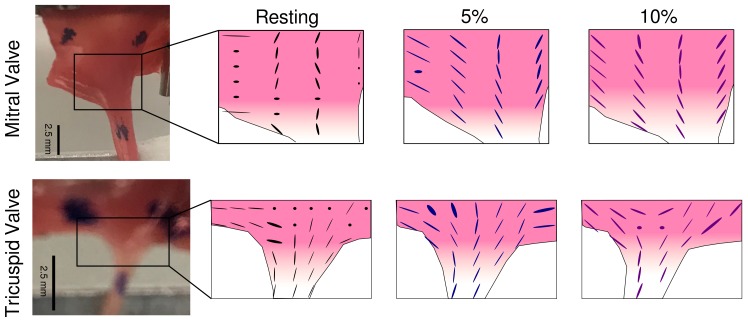
Local collagen fiber alignment calculated from 2-D XRD scans on ‘bisected’ CT–LL samples from TV and LV. The alignment at each datapoint is represented as an ellipse. As is observed with the 5% and 10% stretch series, more fibers become more aligned with increased overall strain applied on the sample.

2D XRD scans were performed on ‘bisected’ CT–LL samples from MV and TV. Local collagen fiber orientations are reported as aligned ellipses with the direction of the major axis pointed along the fiber direction (Figure 3). See MuscleX documentation for details on orientation calculations [35]. The length of the minor axis of the ellipse is inversely proportional to angular spread of diffraction at that location, i.e., the wider the angular (azimuthal) spread of diffraction, the smaller the minor axis of the ellipse. Dots in the map represent locations where isotropic (circular) diffraction patterns were observed indicating the poorer alignment (packing) in these fibers. This behavior changes with the application of stretch, as is evident in the 5% and 10% stretch series, in comparison to the ‘Resting’ state, where fibers in the LL, CT and the transition are compacted, leading to better axial fibrillar packing. This process of change in fiber alignment with the application of stretch provides additional compliance to stretch in these samples, particularly in the LL region.

### 2.4. Microscopic Evaluation of Stress and Strain on Individual Tissue Elements

Individual sample elements, i.e., PM, CT and LL, were stretched continuously until breakage. The stress vs. strain was calculated and is reported as below. The CT assumes most stress and the least amount of strain. This is in line with the functional aspects of the highly tensile tendons. PM assumes most strain for the least stress, with LL lying between the two other elements. The PM to CT mechanical differences are relatively comparable to data from skeletal muscle–junction tissues examined previously Madhurapantula et al. (2017) [13], although the CT–LL observations are clearly distinct from early reports of the muscle–tendon junction.

### 2.5. Microscopic Evaluation of Stress and Strain in Bisected Samples

**Figure 4:**
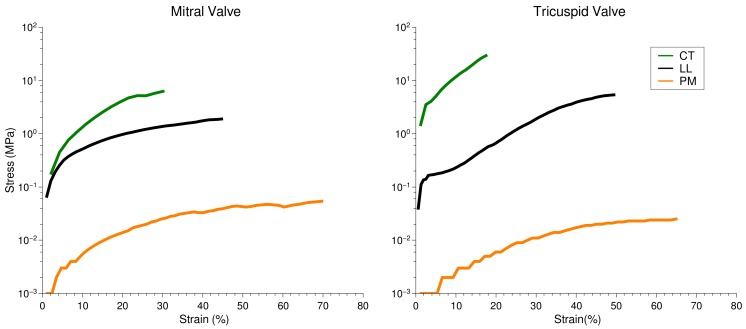
Stress vs. strain evaluation of individual sample elements from MV and TV samples tri-sected so as to measure pure tissue sub-types independently.

While application of stretch on individual tissue elements is important to understand material and mechanical properties, transitions may be understood by performing similar stretch experiments on ‘bisected’ samples that leaves these regions (and surrounding structures) intact. As observed in Figure 4, the CT assumes most stress, while the PM assumes most strain, with LL in between these two strain ranges. The transition regions, however, exhibit markedly different stress vs. strain response than any of the constituent elements. This is expected behavior in light of the different materials forming the interface and the varied packing and are in line with previous observations from Madhurapantula et al. (2017) [13]. The PM–CT transition in both TV and MV exhibits the second highest stress vs. strain curve. This further points to the possibility that this region is relatively the most fragile region and consequently is susceptible to breakage through injury. Some lag is observed in the LL stress both MV and TV, in the ~5% to ~20% strain region. This may be a result of the change in fiber packing and alignment producing multiple ‘toe’ regions in these plots, as shown in Figure 3 (see Section 2.3). 

**Figure 5:**
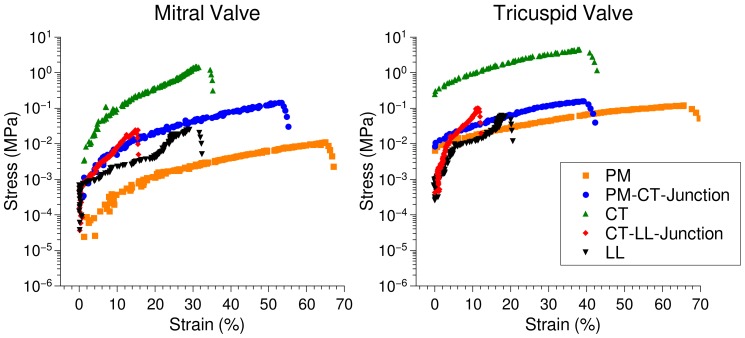
Stress vs. strain evaluation of individual sample segments from MV and TV bisected samples so as to measure these properties in the transition regions in addition to that of pure regions connected to the transition. Stress vs. strain plots were calculated by tracking the movement of fiducial marker. The PM is the most compliant element in the assembly, assuming most strain over the least amount of stress. The CT is the most tensile element, assuming least strain for most stress. The PM–CT junction, in both valves, has the second highest stress for strain. Several points were recorded after the beginning point of sample failure showing the steep decline in the plot of strain vs. stress for each component. These data were used to help determine the ultimate tensile strength (stress) of each tissue component.

**Figure 6:**
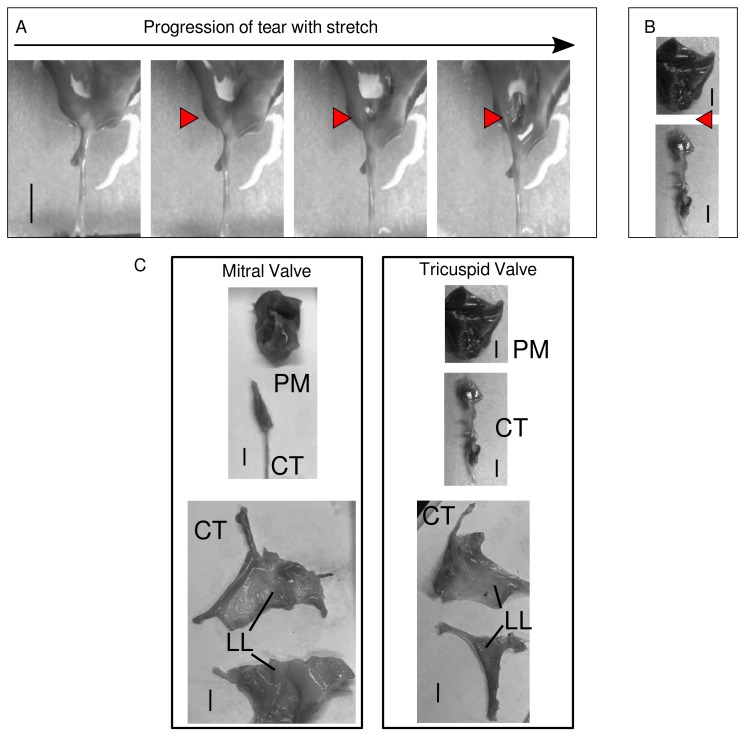
(**A**) Tear in the PM–CT junction of the TV. The red arrowheads show the beginning and progression of the tear in the sample with stretch. (**B**) shows a picture of a torn sample and the point of detachment. (**C**) Post-tear. Note in (**B**) and (**C**), the PM–CT sample from TV were recorded in one image that was modified to place the PM and CT elements closer together for direct comparison to MV image. The sample itself was not modified in the image. Scale bar in all panels shows 5 mm.

As a crude test of valve material vulnerability in the transition regions, sample failure pulls were performed for the MV and TV. Since the CT possesses the ultimate strength of the assembly, it was bisected and the PM–CT and CT–LL assembles tested. Ultimate strength (stress before the sample is permanently deformed) can be determined from Figure 5. These values are recorded in Table 1. The location of the tears in the MV or TV sample were recorded by stretching samples under a video-enabled microscope. Readily observable tears occur at the PM–CT junction of both valves, as seen in Figure 6. The exact location of the tear in the PM–CT sample was recorded by stretching samples under a video-enabled microscope. The tear appears at the PM–CT junction, as seen in Figure 6. This may also indicate multiple levels of tissue hierarchical organization in the LL. The exact point of the tear for the CT–LL was not clearly apparent, possible due to the shape of the LL and its markedly different material behavior (fiber re-arrangement). For both valves, it was not clear if the CT–LL transition region as defined in Figure 2 was the point of failure (not observed in video of pull). Although it may be occurring in the final part of the CT–LL of the MV (Figure 2 and Figure 6), it is not likely our data indicates the same for the TV. It is possible, that there is another internal to the LL transition region of material organization and given its multiple levels of structural hierarchy made principally of the same fibrous material, we were not able to detect a third transition region (if it exists). We note that Figure 2 (particularly part C) indicates a clearer molecular strain relationship in CT–LL to its detected transition for the MV than it does for the TV.

## 3. Discussion

As is evident from the data presented, the PM–CT and the CT–LL transitions present different stress–strain properties in comparison to the ‘pure’ LL, CT and PM sections of the sample (Figure 4 and Figure 5). PM is the most compliant region showing the greatest stress as a function of strain, followed by the LL and CT in this order. A realignment of collagen fibers in the LL regions as observed in Figure 3 leads to a delayed rise in stress with increasing strain (both engineering and molecular strain), in comparison the CT region, which in principle, has a similar composition (collagen and proteoglycan).

The ultimate strength (point on the stress–strain curve marked by permanent deformation) of each region is markedly different (Table 1). This may be explained by the intersection of two significantly different types of material comprising the tissue in the PM–CT region. The transitions between dense collagen arrays and thick muscle fibers in this region to thin muscle and less dense collagen arrays, make it weaker in comparison to the rest of the tissue assembly. Similarly in the CT–LL region, the change in collagen fiber orientation and reduced density of load-bearing CT collagen at the CT–LL transition while the LL component is still of diminished thickness relative to the pure LL region before, gives rise to an ultimate strength that is less than the pure tissue constituent types.

For both the TV and MV, the ultimate stresses within the transition regions (PM–CT and CT–LL) are not much greater than the PM or LL regions (Table 1) even with the stiffer properties of the dense collagenous CT component (Figure 4). However, the compliance of the transition regions, as indicated by Figure 6, is significantly reduced relative to PM or LL. As Figure 2 shows, these same transition regions, experience elevated strain per unit thickness of the tissue region relative to the PM, CT or LL regions that are ‘pure’ and not transitional. Cumulatively, this indicates a mechanical vulnerability to failure relative to the more ‘pure’ parts of the sample: the more compliant but thick PM; the more compliant due to fibrillar re-arrangement capabilities of the LL, and; the materially more durable due to dense construction of relativity non-compliant but strong material (CT). The transition regions have aspects of the ‘pure’ regions, but are vulnerable due to the nature of the transition from stiff to compliant (PM–CT and CT–LL) construction. Figure 6 demonstrates this in showing the mechanical failure in the PM–CT region, exactly where Figure 2 indicates this material vulnerability is most elevated (Figure 2C).

Cumulatively, this indicates a mechanical vulnerability to failure relative to those more compliant but thick (PM) or compliant due to fibrillar re-arrangement (LL) or materially durable due to dense construction of relativity non-compliant but strong material (CT and LL). Figure 6 demonstrates this in showing the mechanical failure in the PM–CT region, exactly where Figure 2 indicates this material vulnerability is most elevated (Figure 2C).

## 4. Materials and Methods

Additional materials and methods are located within the Supplementary Materials, Section S2. No live animals were used for the studies described here. The use of and sourcing of tissues (from 3rd party vendors) were approved by the Illinois Institute of Technology Office of Research Compliance.

### 4.1. Pig Heart Valve Priority and Dissection

Whole, snap-frozen porcine hearts were procured from Carolina Biological Supply (Charlotte, NC, USA; Cat. No.: 228566). The mitral (MV), tricuspid (TV) and the aortic (AV) valves were carefully dissected en bloc to preserve underlying structures (PM, CT and LL). Valves were dissected with enough clearance to ensure that the samples of interest were not damaged during dissection. The dissected valves were then stored at −20 °C. Samples were thawed overnight at 4 °C before experiments.

Clear sections of these valves that consisted of a portion of the leaflet with a singular connection to a CT leading into a PM were identified and dissected. Any branches of the CT were severed, to ensure that there was one main connection between the three tissue elements. The samples were dissected under 1X phosphate buffered saline (PBS) to keep them hydrated during the course of dissection and were stored at room temperature under a paper towel soaked in PBS before experiments. Samples were not stored in PBS buffer longer than 6 h.

Samples were evaluated in three modes: (i) full valve samples (LL to CT to PM); (ii) samples ‘bisected’ by cutting the full valve assembly at the middle of the CT, to evaluate the properties of LL to CT and CT to PM transitions, and; (iii) ‘trisected’ samples, i.e., valve elements separated to evaluate the properties of the LL, CT and PM individually.

It was determined at an early stage of investigation that a more direct comparison of structure and physiological roles of the MV and TV would be the study priority. Due to the anatomical placement of the aortic and pulmonary valves, dissecting both valves from the same heart may compromise one or both of these valves. Moreover, MV and TV present higher complexity in valve architecture than the pulmonary and aortic valves. The pulmonary valve was excluded due to its relatively low-pressure function and because it is rarely reported to fail. Given these priorities, in a number of instances it was viable to retrieve the aortic valve, hence allowing some limited study of these samples also, reported in the SI Section S3. It was not possible to recover viable pulmonary valves after recovery of the higher priority valves.

### 4.2. Custom-Built Tissue Strain Apparatus

**Figure 7:**
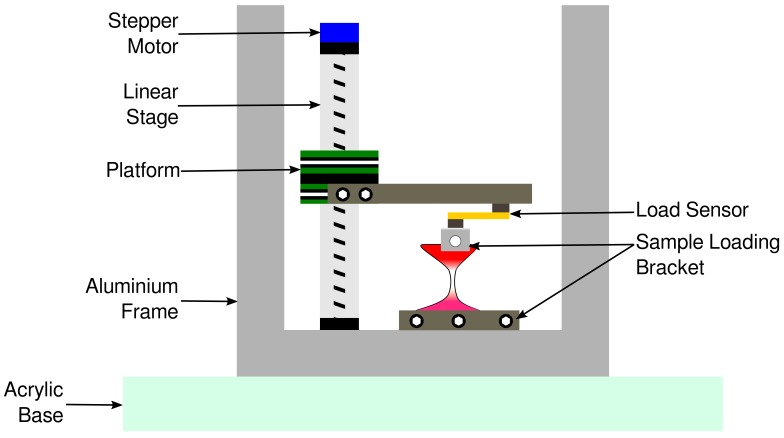
Sample strain apparatus. The stepper motor actuates the stage up to strain the samples loaded between the sample loading brackets. The aluminum frame is attached to a 1/8th inch clear acrylic sheet which acts as a door (not shown in the illustration) to load samples. The thick acrylic base is used to stabilize the rig during data acquisition and also for mounting the rig at the X-ray beamline on an XY sample positioner.

A strain apparatus was constructed based on that previously described in Madhurapantula et al. (2017) [13]. Briefly, a linear stage actuator was attached on frame built from aluminum tubes. An L-shaped aluminum bracket was attached to the stage on the actuator. A load sensor capable of measuring up to 455 g was mounted on to the lower arm of the aluminum bracket, to which a smaller sample bracket was attached (Figure 7).

Samples were mounted between the top and the bottom sample brackets. The stage on the actuator is moved by the stepper motor. An Arduino based program was used to issue commands to stretch the sample. The Arduino also mediates the collection of displacement (mm) vs. load (g) information into text files, which were used for analyses presented here (Figure 7).

### 4.3. XRD Scanning to Determine Tissue Composition

See SI Methods (Section S2) for details on XRD apparatus. Complete valve tissue sections (PM through CT and LL) and ‘bisected’ (cut in the center of the CT, so that the relatively compliant muscle and its transition region could be measured separately from the comparatively stiff CT/LL) samples, were loaded onto the strain apparatus. XRD scans were performed by moving the samples vertically from top (PM) to bottom (LL) with the CT in the middle. Samples were kept wet during the experiment by placing PBS-soaked paper towel pieces in contact with the top and the bottom of the sample and also by squirting PBS using a transfer pipette periodically.

### 4.4. XRD Scanning to Determine Molecular Strain with Application of Stretch

Molecular strain, is defined after Madhurapantula et al. (2017) [13]: for collagen it is the increase in length of the D-period (measured from the 5th order of collagen), which is a measure of molecular/fibrillar level length increase in the collagen molecules due to straightening of the tilted molecules [36,37,38]. All data analysis was performed using MuscleX [35].

For muscle, it is the increase in sarcomere length which is directly related to lengthening of the muscle fibers due to myosin and actin fiber sliding in response to an application of stretch [13]. Muscle fiber elongation has been shown to be related to the inter-filament spacing, specifically the observation that the d-spacing of the muscle equatorial (1,1) reflection has a clear relationship to sarcomere length [39]. However, as described in Yagi et al. (2004) [39], this observation shows some variation, possibly based on sample storage and preparation. Therefore, for this study, the change in the d-spacings of the (1,1) reflection are interpreted as a relative, approximate, measure of fiber elongation (molecular strain) in contrast to the more accurate and reliable, D-period change in collagen measurements. The muscle ‘molecular strain’ is included in the SI Section S3.1 for comparison to that data obtained for collagen ‘molecular strain’. Engineering strain (or strain) is defined as the percentage change in length of the tissue sample (whole tissue or region of the whole tissue), upon application of stretch.

2D scans were performed on bisected samples to determine changes in alignment of collagen fibers in the PM–CT and the CT–LL junctions with application of stretch. The initial sample measurements were noted and stretch was applied to the samples at increments of 2% engineering strain. The sample was allowed to relax for 2 min before 10 XRD patterns were collected along the central sections of the LL–CT and CT-PM samples.

### 4.5. Microscopic Evaluation of Valve Components

**Figure 8:**
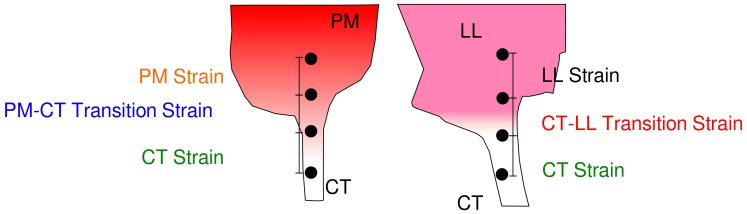
An illustration of location of fiducial markers placed on samples for tracking using the TrackMate plugin on Image J. Markers are placed so that each region (PM, CT and LL) have at least have one marker and one on each side of the visible transition in each sample type.

‘Bisected’ valve samples (LL–CT; CT-PM) were loaded on to the strain rig described herein. Visual markers (‘dots’) were placed on the sample using a marking pen (Sharpie™). These dots were used as fiducial markers. These markers were placed so there was at least one each in the PM or LL and a pair of dots on either side of the transition region (Figure 8). The elongation in the transition region was used to calculate local strain in the transition region, for comparison with overall sample strain. Samples were stretched using the computer interface, to reach maximum stress. The sample was visualized using a standard point and shoot camera (Kodak™ PIXPRO FZ53) and video sequences were recorded for the entire length of the application of stretch.

The video was then used to generate still frames at 5 fps. These frames were imported into ImageJ [40] for analysis with the TrackMate plugin [41]. This plugin generates a frame-by-frame X and Y position of each marker at each frame. These positions were then used to calculate the distance between the various markers in each frame, and hence the strain, by fixing the distances from the first frame as starting lengths for each region. Stress was calculated from the load recorded by the strain apparatus and using the cross-sectional area of each region. Additionally, once transition regions were defined for each sample type, new samples were ‘trisected’ for clearly defined regions of PM, CT and LL so that the possible transition regions were removed from measurements of ‘pure’ tissue components, for comparison.

## 5. Conclusions

Although it is often reported that CT is the point of valve failure, our data indicate that the definition of which portion of the valve is CT is highly relevant to the material outcomes of the tissue. Although the CT tissue is present through transition regions from the PM to CT and from the CT to LL, its durability through these material regions is different from that of the CT main section. The significance of tissue transition regions have often been overlooked, when studying mechanical behaviors of soft tissues. Emphasis is usually laid on understanding the individual tissue components. However, as is evident from earlier surgical observations [42,43] and data presented here, transition regions show substantially different material and mechanical properties from the ‘pure’ tissues at their boundaries. Notably, the transition regions should be considered as strong candidates for regions in which mechanical failure predominantly occurs, as has been reported previously [44,45]. These large material composition and mechanical differences through the transition regions may be highly relevant to understanding valve failure and in finding remedies for it.

The methods and data presented here allow attribution of material properties to these regions and their behavior under strain. XRD scans enable determination of high-resolution tissue composition in transition regions in their native state (ex vivo) without introducing possible artifacts due to sample preparation (fixing, staining, affixing fiducial markers by stitching/gluing, etc.) or in downstream data preparation where 2D sample image sections need to be reconstructed into a 3D volume before detailed analysis is performed. The molecular strain analyses in the context of engineering strain, in relation to the tissue composition, provide insights into changes in the molecular packing of materials in samples that lead to strain distribution in these regions in relation to its change in material composition as it transitions from one tissue into another. No other technique, to our knowledge, allows similar static and dynamic analyses of the simultaneously multi-scale properties (nanoscopic through to large-scale mesoscopic) collected from a native untreated sample while it is stretched.

Studies have shown that virtual reality-based technique used to train surgeons are highly beneficial [46,47]. The methodology developed in this study may well be used to advance the state and biofidelity of simulators for surgical simulators and also to improve tool–tissue interactions in robot-assisted surgery (and planning), since these data enable definitions that are likely significantly closer to tissue behavior in vivo.

## Figures and Tables

**Table 1:** Ultimate tensile strength (stress) determined from microscopy data of bisected samples reported in Figure 5. These peak stress values were reached by these regions before the samples were permanently deformed by application of stretch.

	Ultimate Stress (MPa)	
	Mitral Valve	Tricuspid Valve
PM	0.011	0.119
PM–CT	0.144	0.159
CT	1.491	4.503
CT–LL	0.024	0.097
LL	0.025	0.059

## Supplementary Materials

The following are available online at https://www.mdpi.com/1422-0067/21/3/763/s1.

## Abbreviations

PM	Papillary Muscle
CT	Chordae Tendinae
LL	Leaflet
MV	Mitral Valve
TV	Tricuspid Valve
AV	Aortic Valve
PG	Proteoglycan
XRD	X-ray Diffraction
