# Supplementary material for: Advanced Methodology and Preliminary Measurements of Molecular and Mechanical Properties of Heart Valves under Dynamic Strain
Source: Int J Mol Sci. 2020 Jan 24;21(3):763. doi: 10.3390/ijms21030763 (PMC7037596; doi:10.3390/ijms21030763)
Supplement: Supplementary file 1 [file ijms-21-00763-s001.pdf]

International Journal of *Molecular Sciences*

MDPI

Supplementary Materials: Advanced Methodology and Preliminary Measurements of Molecular and Mechanical Properties of Heart Valves Under Dynamic Strain

Rama S. Madhurapantula[1,2,*], Gabriel Krell[2], Berenice Morfin[1], Rajarshi Roy[3], Kevin Lister[3], Joseph P.R.O. Orgel[1,2,4,*]

S1. Background

S1.1. Sarcomere Lengths of Skeletal and Cardiac Muscles

The sarcomere is the basic repeating unit in a striated muscle fiber. It is defined as the distance between two adjacent Z-lines, as seen Figure S1. The muscle fiber lengthening is directly related to increase in sarocmere length (SL). Papillary muscles (PM) arise from and are closely (anatomically) associated with cardiac muscles, while their SL has been reported to have a similar range to that of skeletal muscle. A comparison of SL of different muscles from various organisms is presented in Table S1.

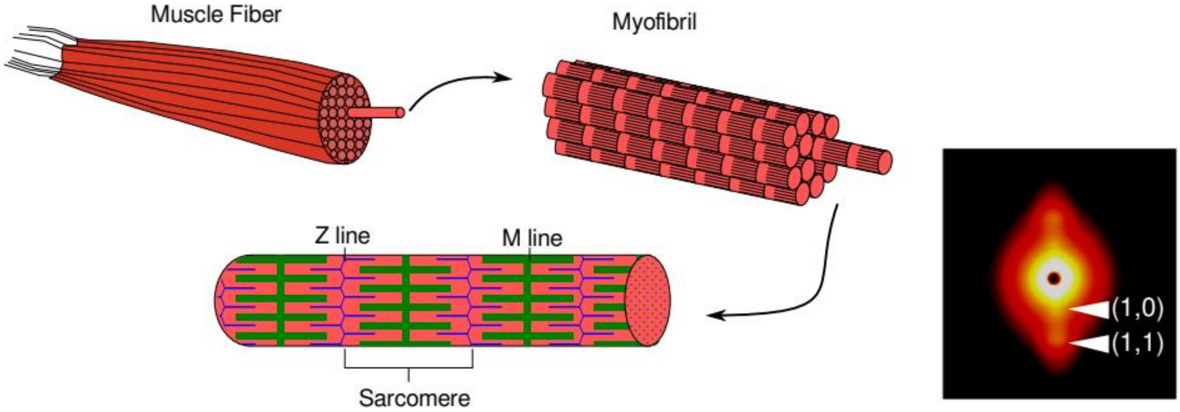

Figure S1. Illustration of the hierarchical packing structure in a muscle fiber. Sarcomere length may be determined using the X-ray diffraction pattern shown on the right side of the figure. The (1,0) and (1,1) equatorial reflections were used to track strain and composition in valve tissues.

Table S1. Sarcomere lengths of papillary, skeletal and cardiac muscles from various animals. The respective works reporting these values are indicated.

	Muscle Sarcomere Length (µm)		
**Animal**	**Papillary (Cardiac)**	**Skeletal**	**Cardiac wall (Cardiac)**
Cat	2.3-2.6 [48]	2.0-2.4 [49]	1.58-2.15 [50]
Rabbit	2.34-2.45 [51]	2.2-3.4 [52]	1.71 [53]
Rat	2.23 [54]	2.26-2.43 [55]	1.6-2.2 [56]
Pig	2.1-2.3 [7]	2.35-3.07 [6]	1.4-2.2 [8]

S1.2. Collagen hierarchical organization

The packing and organization of type I collagen in situ is complex. Several works on the packing structure of collagen in rat tail tendons have been a wealth of information on the molecular structure function relationships and ligand binding activity [36–38]. Changes in this molecular

packing have directly been correlated to various diseases. For the experiments and interpretations presented here, the changes in the D-period (molecular strain) have been tracked using the diffraction pattern from type I collagens.

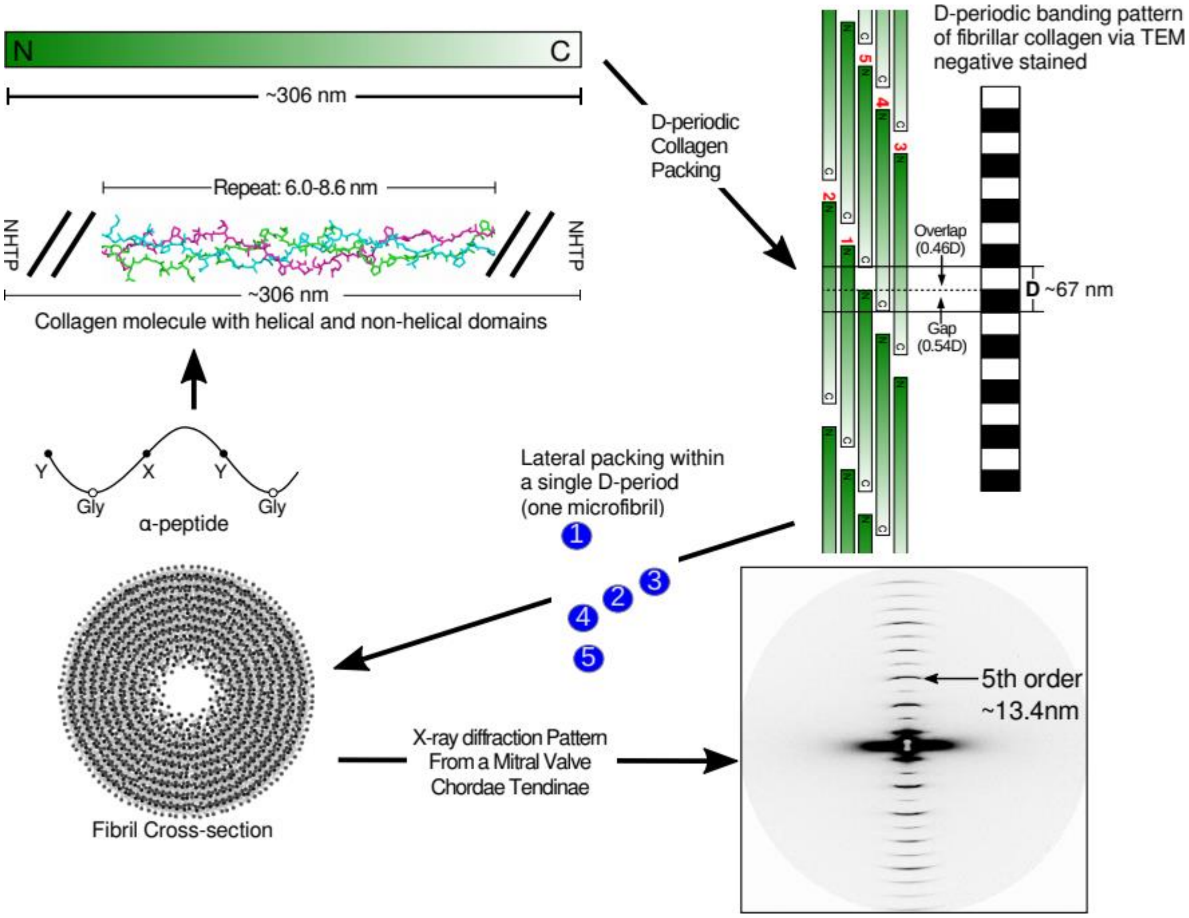

Figure S2. Hierarchical structure of collagen in structure of collagen in fibrillar tissues. The monomers of collagen consist of an ~306 nm long triple helix that is composed of peptides made by a Glycine-X-Y amino acid repeat. Proline and hydroxproline commonly occupy the X and Y positions but the primary sequence is still around 50% amino rather than immino acid. The triple helical regions are flanked on either ends (N and C- terminus) by non-helical telopeptides (NHTP). These monomers are arranged into a three dimensional array where the neighboring collagen molecule are staggered along the fiber axis to form the collagen microfibril. This 3D packing structure gives rise to the signature ~67 nm D-periodic arrangement, consisting of one overlap region where 5 monomers segments align and the gap region, where only 4 monomer segments align. The length of the D-period can be measured using the 5th order d-spacing from the X-ray diffraction pattern. Increase in D-period length with the application of stretch, relative to that recorded in resting state, is reported as molecular strain. The lateral packing in this illustration is for type I collagen from rat tail tendons. Chordae tendinae are predominantly made up of type I collagen and are a fibrillar soft connective tissue. Hence, the lateral packing can be assumed to be similar to that type I collagen from rat tail tendons. Illustration adapted from Orgel et al., 2006. [36] and Orgel et al., 2014 [38].

S1.3. Leaflet Composition and Layers

The leaflets of MV and TV are composed of several layers of materials, that contribute to elastic property and function. Principally, the leaflets consist of three different types of collagen fibers, the most predominant being type I collagen at 74%, followed by type III at 24% and type V at 2% [9]. As described in main text, changes observed in local collagen fiber alignment may be a result of rearrangement of fiber and other components in these layers. McCarthy et al., 2010 [9] described

these various layers, which are illustrated below. Changes to packing and orientation of collagen fibers in these layers explain the delay in the rise of strain with increasing stress in leaflet samples (Main text Fig. 3).

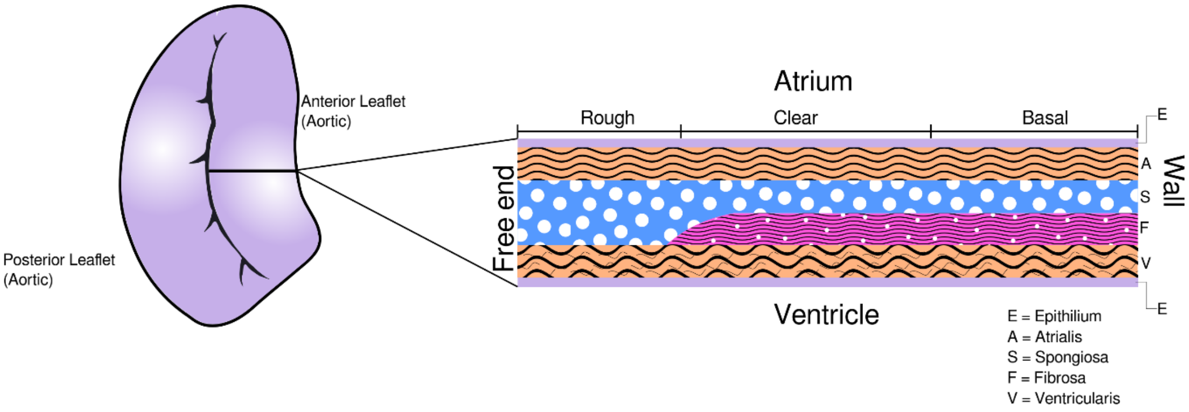

Figure S3. Layers of materials found in valve leaflets. The illustration on the right shows layers of various materials that constitute the thickness of the leaflet between the free end, and the wall of the aorta. The leaflets are lined by epithelial tissues. The atrialis (uppermost layer) consists of elastic and collagen fibers. The spongiosa consists of proteoglycans (PG), and glycosaminoglycans (GAG) which are responsible to attract water into the leaflet, and also swelling of the leaflet on the free end. Fibrosa is the major load-bearing layer of the leaflet, and consists of compacted and aligned collagen fibers. The ventricularis consists of more elastic fibers, which is lined by a layer of epithelium.

S2. Methods

S2.1. X-ray Diffraction Setup

A small angle X-ray Diffraction (XRD) apparatus was used for composition and strain testing at the Biophysics Collaborative Access Team (BioCAT) at the Advanced Photon Source at Argonne National Laboratory, Chicago IL (Fig. S4). A 12 keV (1.033Å) X-ray beam was focused using a vertical focusing mirror to deliver a focused beam (width = 150μm, height = 30μm; flux= ~ 1x1013 photons/s) beyond the sample positioner. A ~4m evacuated flight tube was used to deliver transmitted and diffracted X-rays to a marCCD 165 detector (Rayonix, LLC Evanston, IL) to acquire diffraction patterns. The custom-built strain rig was bolted to a motorized XY-positioner. These motors were used to move the rig to align the sample area of interest with the beam path. Scanning was performed using scripts provided by the BioCAT user facility.

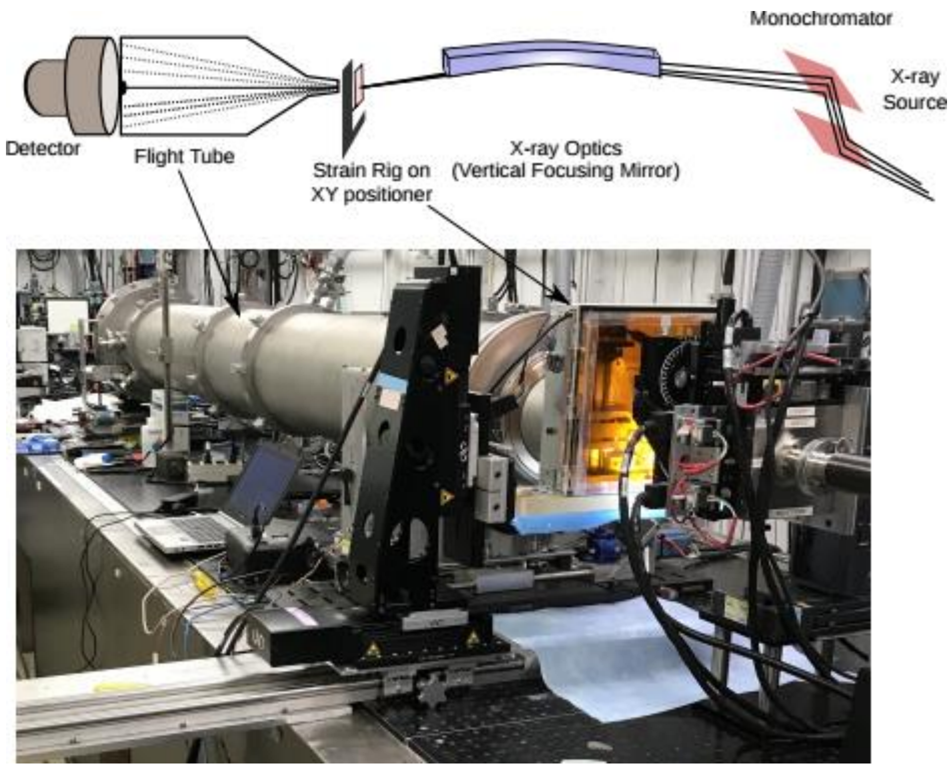

Figure S4. The schematic and a photograph of the XRD setup at BioCAT. The lower panel shows position of the sample strain rig positioned in the beamline for X-ray data collection.

S2.2. Number of Samples

The data presented here are a representative dataset collected from several samples. All samples for each type of analysis were procured from the same heart, to maintain consistency. The total number of samples used are presented in a table below (Table S2). However, each diffraction pattern is cylindrical average of millions of tissue elements. This mitigates the need for a large number of samples to be scanned using X-rays for statistical purposes.

Table S2. Number of samples used for each type of experiment for this study.

Experiment	Number of samples
Bisected Molecular Strain	3 per junction
Bisected Microscopic Strain	3 per junction
Trisected Microscopy	4 per sample section

S3. Results

S3.1. Changes in Muscle Molecular Strain in the PM-CT Junction

It is evident from microscopic testing that PM is the least tensile (assumes most strain). Approximate muscle molecular strain (see comment in methods) may be calculated by tracking equatorial muscle reflections, as denoted in Fig. S1. However, since no muscle diffraction is observed in the CT region, these reflections can only be tracked from the PM through the PM-CT junction (Fig. S5). The molecular strain per unit thickness plot (Fig. S5C) shows a localization of peak strain at datapoint 4, which aligns with similar data reported for collagen in main text Fig. 4. This further points to this part of the tissue assembly being at high risk for mechanical (traumatic) failure.

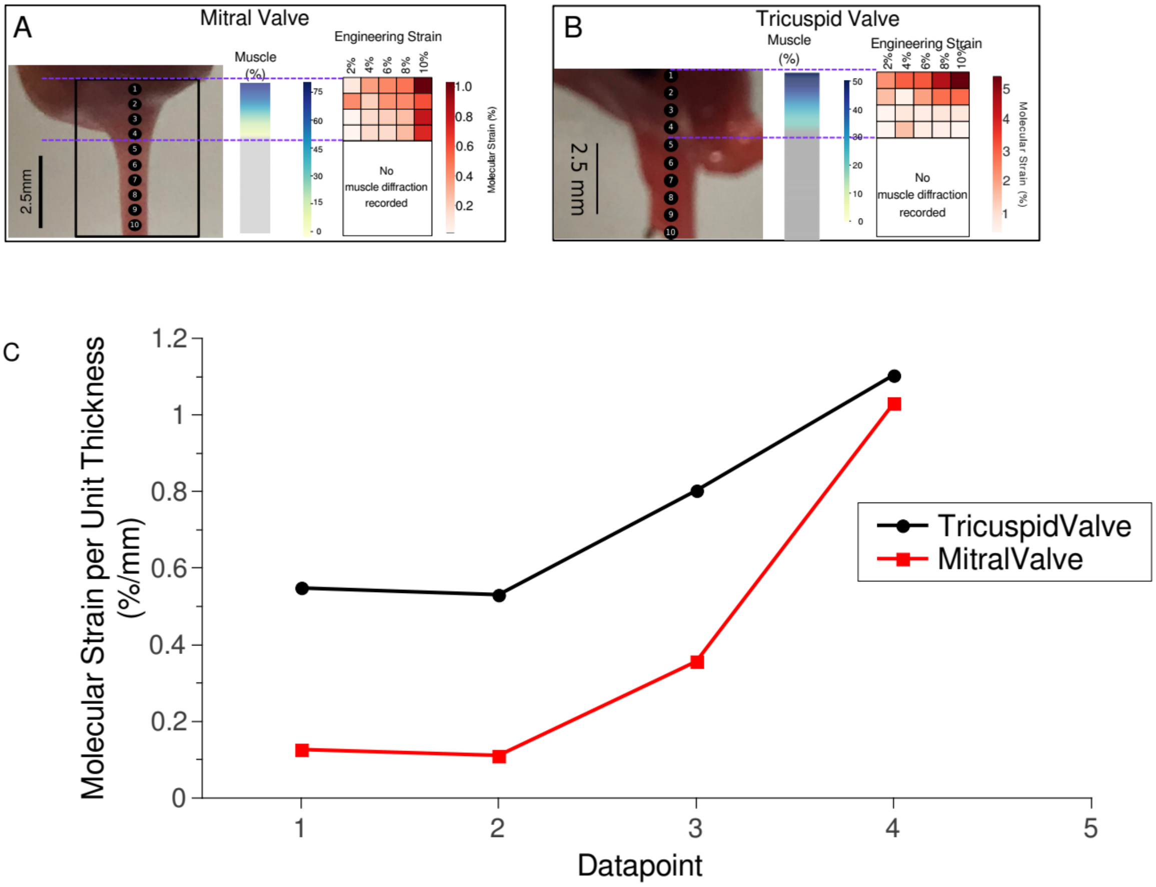

Figure S5. Changes to muscle molecular strain in the PM-CT region. The heatmaps (A & B)show an increase in molecular strain in the PM region of the sample, with a gradual drop through the PM-CT junction. Molecular strain normalized for sample thickness at the location (C) shows that peak strain is observed at datapoint 4, which aligns with the visible PM-CT junction.

S3.2. Aortic Valve Composition and Molecular Strain Maps

The aortic valve (AV) presents one transition region where the leaflet (LL) attaches with the wall of the aorta and muscle. This muscle to leaflet transition was tracked using XRD scanning, as reported for mitral and tricuspid valves. The LL to wall samples were also stretched from 2-10% stretch points at 2% increments. Molecular strain data was reported as heatmaps.

The AV presents one highly diffuse and not immediately obvious (from visible inspection) transition from PM to 'CT' and possible a second slight transition from 'CT' to LL, unlike MV and TV where these transitions are evident and both the CT and LL are distinctive. The data presented here show two regions in the samples where molecular strain is localized with stretch application. The first region is observed along the visible junction between the muscle and 'CT'/LL side of the CT of the sample. Muscle diffraction disappears within 0.4 mm in the muscle to LL transition. However, the localization of strain in this region resembles that observed in MV and TV. A second region of strain localization can be observed on the muscle side of the sample. This is also evident in the molecular strain per unit thickness as seen in the bottom 1D plot Fig. S6. This localization of strain is a result of the reduction of muscle thickness in this region.

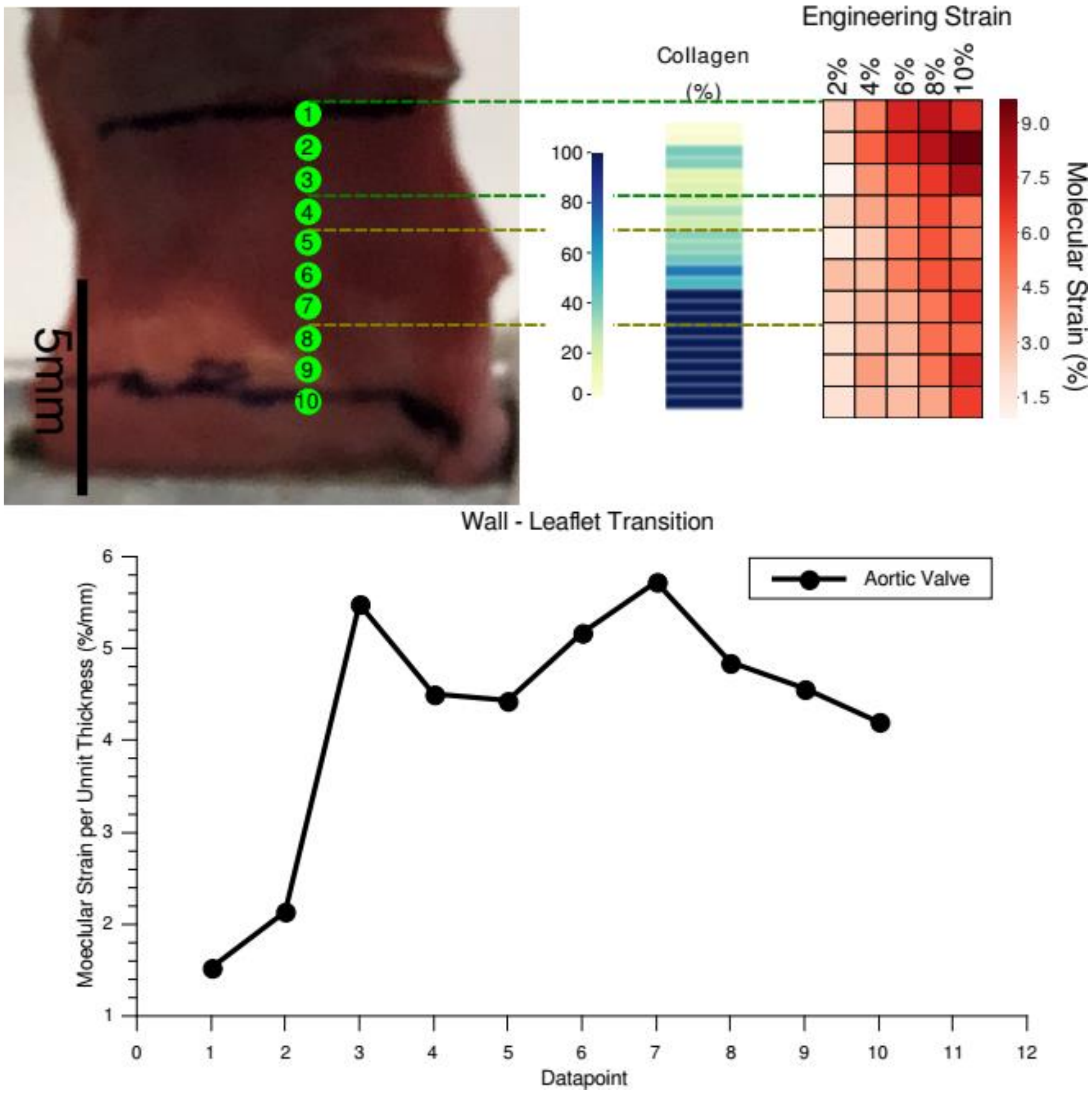

Figure S6. Composition and molecular strain maps from a porcine aortic valve. The composition maps show relative collagen percentage along the wall (muscle) to leaflet transition in comparison to muscle. The green dotted line shows a second region of strain localization. Within this region, an overall increase in strain is observed between datapoints 2 and 3. The region marked by olive dotted lines aligns with the visible boundary between the two tissue types. There is an overall increase in strain with stretch application in this region, yet not as much as that observed in the upper region marked by the green dotted lines. Molecular strain per unit thickness, plotted at 10% engineering strain also indicates the two regions of strain localization.

References

48. Fawcett, D.W.; McNutt, N.S. The Ultrastructure of the Cat Myocardium. *J. Cell Biol.* **1969**, *42*, 1–45, doi:10.1083/jcb.42.1.1.
49. Vaz, M.A.; de la Rocha Freitas, C.; Leonard, T.; Herzog, W. The force-length relationship of the cat soleus muscle. *Muscles Ligaments Tendons J.* **2012**, *2*, 79–84.
50. Keurs, H.E.; Runsburger, W.H.; van Heuningen, R. Restoring forces and relaxation of rat cardiac muscle. *Eur. Heart J.* **1980**, *1*, 67–80, doi:10.1093/eurheartj/1.suppl_1.67.
51. Wohlfart, B.; Grimm, A.; Edman, K. Relationship between Sarcomere Length and Active Force in Rabbit Papillary Muscle 1. *Acta Physiol. Scand.* **1977**, *101*, 155–164.
52. Horowits, R. Passive force generation and titin isoforms in mammalian skeletal muscle. *Biophys. J.* **1992**, *61*, 392–398.

53. Torre, I.; González-Tendero, A.; García-Cañadilla, P.; Crispi, F.; García-García, F.; Bijnens, B.; Iruretagoyena, I.; Dopazo, J.; Amat-Roldán, I.; Gratacós, E. Permanent Cardiac Sarcomere Changes in a Rabbit Model of Intrauterine Growth Restriction. *PLoS ONE* **2014**, *9*, e113067, doi:10.1371/journal.pone.0113067.
54. Julian, F.J.; Sollins, M.R. Sarcomere length-tension relations in living rat papillary muscle. *Circ. Res.* **1975**, *37*, 299–308, doi:10.1161/01.res.37.3.299.
55. ter Keurs, H.E.D.J.; Luff, A.R.; Luff, S.E. Force—Sarcomere-Length Relation and Filament Length in Rat Extensor Digitorum Muscle. In *Advances in Experimental Medicine and Biology*; Springer: New York, NY, USA, 1984; pp. 511–525, doi:10.1007/978-1-4684-4703-3_44.
56. de Tombe, P.P.; ter Keurs, H.E.D.J. Cardiac muscle mechanics: Sarcomere length matters. *J. Mol. Cell. Cardiol.* **2016**, *91*, 148–150.
